# An Atypical Squamous Papilloma of the Uvula

**DOI:** 10.7759/cureus.58008

**Published:** 2024-04-10

**Authors:** Afena Apandi, Lum Sai Guan, Amran Mohamad, Fatin Muhamad Tamyez, Muhammad Nu’aim Ishak

**Affiliations:** 1 Department of Otorhinolaryngology - Head and Neck Surgery, Faculty of Medicine, Universiti Kebangsaan Malaysia, Kuala Lumpur, MYS; 2 Department of Medicine, Hospital Canselor Tuanku Muhriz Universiti Kebangsaan Malaysia (UKM), Kuala Lumpur, MYS; 3 Department of Otorhinolaryngology - Head and Neck Surgery, Faculty of Medicine, Universiti Kebangsaan Malaysia (UKM), Kuala Lumpur, MYS; 4 Department of Otorhinolaryngology, Hospital Sultanah Nur Zahirah, Kuala Terengganu, MYS; 5 Department of Pathology, Hospital Sultanah Nur Zahirah, Kuala Terengganu, MYS; 6 Department of Otorhinolaryngology - Head and Neck Surgery, Universiti Sultan Zainal Abidin, Kuala Terengganu, MYS

**Keywords:** warts, treatment outcome, soft palate, sensation, foreign bodies, human papillomavirus viruses, papilloma, squamous cell

## Abstract

Squamous papilloma of the oral cavity is frequently seen in adult patients and is typically presented as painless exophytic granular or cauliflower-like lesions over the tongue, floor of the mouth, palate, uvula, lips, and faucial pillars. Most of the lesions are solitary and grow rapidly to about 0.5 cm. Oral squamous papilloma has no known malignant potential, with conservative surgical excision being the treatment of choice. Recurrence is rare. It occasionally causes symptoms, unless the presentation is atypical, as in our case. An elongated uvula can cause discomfort and reduce a patient's quality of life. This study aims to report an atypical presentation of a squamous papilloma over the soft palate.

## Introduction

This paper has been accepted for the e-poster competition of the 23rd Medical and Health Research Week, and the abstract was accepted and displayed under the clinical category of the Medicine and Health Journal’s Special Issue on September 20, 2021. The World Health Organization (WHO) defines squamous cell papilloma as a benign hyperplastic exophytic localized proliferation with a verrucous or cauliflower-like morphology. It can occur at any age, but predominantly in the third to fifth decades of life, with no gender preponderance [1]. Squamous cell papilloma is the fourth most common oral mucosal mass and represents 3% to 4% of all biopsied oral lesions [2]. It consisted of 2.5% of all oral lesions [3]. The papillomas arise after infection due to various types of noncarcinogenic human papillomavirus (HPV), which result in benign epithelial proliferations containing the virus in an episomal state [3]. The most commonly reported HPV infection types are 6 and 11 [1]. The average size of the oral papilloma was less than 1.0 cm, and only 8% were 2.0 cm [4]. Most of the studies reported a squamous papilloma size of less than 1.0 cm, and only 7.8% of them measured between 2.0 and 3.0 cm [5,6]. Brady et al. reported a case of hypoxemia during conscious sedation due to partial airway obstruction by a pedunculated benign squamous papilloma of less than 1.0 cm [7]. It is important to recognize the uvula abnormality and give appropriate management. We present a rare occurrence of a squamous papilloma that arises from the uvula and grows tremendously long.

## Case presentation

A 51-year-old Malay male, married and an active smoker, complained of a foreign body sensation in the throat for one week. Before that, he had throat discomfort for two years but never sought medical advice. The patient noticed his uvula was progressively growing in size, reaching the anterior part of his tongue. He had no symptoms suggestive of laryngeal involvement, such as a change of voice, aspiration, or noisy breathing. He had no other symptoms such as odynophagia, dysphagia, blockage of the nose, epistaxis, fever, loss of weight, or loss of appetite. He had no other risk factors, such as high-risk behavior or a history of trauma. He also had no medical comorbidities.

**Figure 1:**
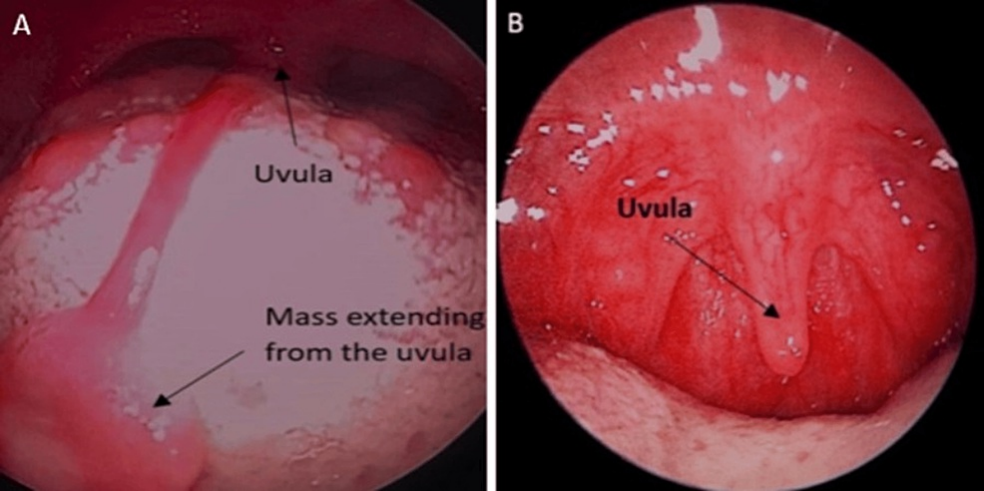
Oral cavity. (A) The extremely elongated soft tissue mass arising from the tip of the uvula, lying on the tongue surface. (B) Normal uvula post-excision of the lesion.

**Figure 2:**
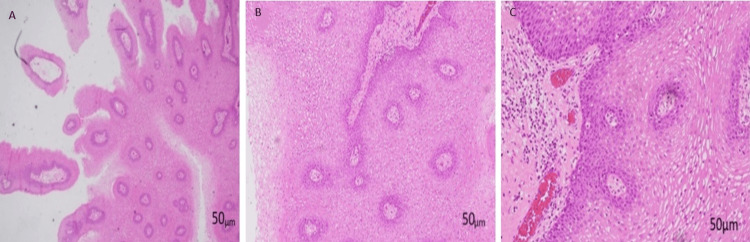
Histopathology examination of the mass was reported as squamous papilloma. (A) Exophytic lesion with arborizing fronds; (B) lined by thickened, mature squamous epithelium; and (C) occasional koilocytosis, no atypia.

An oral examination revealed an elongated strand of soft tissue arising from the tip of the uvula, extending to the anterior one-third of the tongue. The mass measures about 5.0 cm in length with an exophytic granular end, as shown in Figure 1A. There was no bleeding upon touch, and it was nontender. It was soft and consistent. Bilateral tonsils were grade I with normal mucosa. No other abnormal mass was seen in the oropharynx. There was no palpable neck node. In a clinical setting, the uvula mass was successfully excised using local anesthesia without complications. Adequate local anesthesia was infiltrated, and the uvula was clamped for one minute and excised using blade 15. Bleeding was secured using diathermy. The patient followed up a week later, as shown in Figure 1B. The histopathology examination of the mass was reported as squamous papilloma (Figure 2). It shows the exophytic tissue composed of arborizing papillary fronds with a fibrovascular core (Figure 2A) and consisting of thickened and matured squamous epithelium (Figure 2B). The basal layer is hyperplastic, with occasional koilocytosis (Figure 2C). No atypia or evidence of malignancy is observed. During the subsequent follow-up, the patient was well, with no recurrence at the one-month follow-up. The foreign body sensation completely disappeared.

## Discussion

Squamous papilloma is characterized by painless exophytic granular to cauliflower-like lesions that are mostly caused by non-carcinogenic HPV infection [3]. Tomes first described the lesion as a gingival *wart* in 1848 [5]. The papilloma may be pedunculated or sessile. The pedunculated lesions are composed of a cluster of finger-like fronds and may be white or mucosal in color, depending on the degree of keratinization. The sessile lesions are dome-shaped and have a more nodular, papillary, or verrucous surface [1].

Most of the oral papilloma lesions are solitary and about 0.5 cm in size [1]. In certain conditions, multiple papillomata can be seen in the setting of a solid organ transplant or human immunodeficiency virus (HIV) infection [1]. The majority of patients are female, ranging in age from four to 65 years. Most of the studies reported a squamous papilloma size of less than 1.0 cm, and only 7.8% of them measured between 2.0 and 3.0 cm [6,7]. In our case, the oral squamous papilloma grew rapidly to 5.0 cm in length, causing a severe foreign body sensation in the throat. Oral papillomas are benign and generally harmless, but they are potentially hazardous in rare instances. Brady et al. reported a case of hypoxemia during conscious sedation due to partial airway obstruction by a pedunculated benign squamous papilloma of less than 1.0 cm [8].

The histopathology examination of a squamous papilloma typically shows papillary proliferations of the hyperplastic stratified epithelium that are either covered by a layer of parakeratin or orthokeratin of variable thickness or are non-keratinized. The finger-like epithelial projections extend from a narrow base, supported by fibrovascular cores containing dilated capillaries. The stroma may be edematous or hyalinized. Koilocytes are infrequent and mitotic activity is unusual, except in the setting of trauma or inflammation [1].

Most of the etiology of oral squamous papilloma is due to HPV. Carneiro et al. reported the presence of koilocyte-like cells in the specimens, presumed highly suggestive of viral infection [7]. Polymerase chain reaction (PCR) or in situ hybridization can detect the presence of HPV in the lesion [7]. In situ hybridization can detect HPV deoxyribonucleic acid (DNA) using radioisotope-labeled specific probes; however, it is less sensitive compared to PCR [7]. The DNA sequences of HPV 6 and HPV 11 have been detected in the majority of oral papillomas [7]. In cases of recurrent respiratory papillomatosis, HPV 6 and HPV 11 DNA were detected, as in papilloma cases [9]. In adults, co-infection is rare and usually caused by only one of the viruses, while children with laryngeal papillomatosis can have co-infection with HPV 6 and HPV 11 [10].

The differential diagnoses of oral papilloma are verruca vulgaris, condyloma acuminatum, and multifocal epithelial hyperplasia, which are difficult to distinguish clinically [1]. Condyloma acuminatum and verruca vulgaris are both described as exophytic growth patterns. However, histologically, they can be differentiated as condyloma acuminatum by hyperplastic squamous proliferation associated with fibrovascular cores, exophytic growth, and a broad base, while verruca vulgaris is an elongated rete ridge that converges toward the center with a prominent granular cell layer with keratohyalin granules and koilocytic changes [1]. The features of both pathologies almost mimic the squamous oral papilloma presentations; thus, histopathology findings will give diagnostic results.

Surgical excision is the mainstay of treatment, and the prognosis is good and improves quality of life. Oral squamous papilloma is treated with surgical excision under local anesthesia. Our patient had immediate relief after the mass was excised in the office setting at the first consultation visit. The excision was straightforward and simple, well-tolerated, with minimal pain experienced by the patient. Recurrence is uncommon. There have been no reports of malignant transformation or dissemination [1].

## Conclusions

Oral squamous papilloma is a benign tumor that is mostly asymptomatic and can be managed by surgical excision in an office setting. It occasionally causes symptoms, unless the presentation is atypical, as in our case. The size of the lesions may vary individually, but the symptom that arises would lead them to seek treatment. Clinicians should be aware of atypical presentations of squamous papillomas, as this will guide proper diagnosis and management. Implementing appropriate management strategies can significantly improve the patient's well-being.
